# Rothia mucilaginosa Prosthetic Valve Endocarditis Presenting as a Thrombotic Microangiopathy Mimic With Catastrophic Intracranial Hemorrhage

**DOI:** 10.7759/cureus.111711

**Published:** 2026-06-29

**Authors:** Katherine E Guardado, Matthew Faherty, Jonathan Lin, Samuel Wagner

**Affiliations:** 1 Internal Medicine, Mount Carmel Health System, Grove City, USA; 2 Internal Medicine, Mount Carmel Health System, Grove CIty, USA

**Keywords:** anca associated vasculitis, chondritis, diagnostic delay, macrocytic anemia, vexas syndrome

## Abstract

*Rothia mucilaginosa* is an uncommon but increasingly recognized opportunistic pathogen capable of causing invasive infection, particularly in patients with prosthetic material and immunocompromised states. Rarely, severe systemic infections may present with thrombotic microangiopathy-like syndromes mimicking thrombotic thrombocytopenic purpura (TTP), creating significant diagnostic uncertainty.

We present a case of fulminant *Rothia* mucilaginosa prosthetic valve infective endocarditis initially presenting as a TTP/disseminated intravascular coagulation (DIC) mimic in a 60-year-old man with severe thrombocytopenia, schistocytosis, hemolytic anemia, encephalopathy, and intracranial hemorrhage. Due to concern for evolving TTP, emergent plasmapheresis and corticosteroids were initiated prior to identification of the underlying infectious process. Blood cultures subsequently grew *Rothia* mucilaginosa, and transesophageal echocardiography demonstrated prosthetic valve endocarditis. The patient’s course was complicated by progressive bilateral intracranial hemorrhage, septic emboli, and catastrophic neurologic deterioration despite aggressive multidisciplinary management.

This case highlights the diagnostic challenge of distinguishing primary TTP from infection-associated thrombotic microangiopathy and emphasizes the importance of maintaining a broad differential diagnosis in patients presenting with thrombocytopenia, schistocytosis, neurologic dysfunction, and sepsis physiology, particularly in the setting of prosthetic cardiac valves.

## Introduction

Systemic infections may rarely present with thrombotic microangiopathy (TMA)-like syndromes characterized by thrombocytopenia, microangiopathic hemolytic anemia, schistocytosis, and end-organ dysfunction, closely mimicking thrombotic thrombocytopenic purpura (TTP) or disseminated intravascular coagulation (DIC) [1]. Distinguishing true immune-mediated TTP from infection-associated TMA is critical, as delays in recognition of the underlying infectious process may result in rapid clinical deterioration, septic embolization, hemorrhagic complications, and death [1]. Infective endocarditis represents an important but underrecognized cause of pseudo-TTP presentations due to overlapping hematologic and inflammatory manifestations [1]. Mechanistically, severe infection can mimic TTP through diffuse endothelial injury, inflammatory cytokine release, platelet activation, and microvascular thrombus formation, resulting in thrombocytopenia and microangiopathic hemolysis [1]. Concurrent activation of the coagulation cascade may also produce DIC-like findings, including prolonged coagulation studies, hypofibrinogenemia, and elevated D-dimer, further increasing diagnostic overlap [1].

*Rothia* species are Gram-positive organisms that colonize the oral cavity and upper respiratory tract [2,3]. They are increasingly recognized as opportunistic pathogens capable of causing invasive disease, particularly in immunocompromised patients and those with prosthetic material [2,3]. Although uncommon, *Rothia* infective endocarditis has been associated with substantial morbidity, including systemic embolization, neurologic complications, intracranial hemorrhage, and mycotic aneurysm formation [4-7]. In a systematic review of 51 reported cases of *Rothia* infective endocarditis, neurologic complications occurred in approximately one-third of patients, while prosthetic valve involvement was identified in 16% of cases [5]. *Rothia* *mucilaginosa* infections have been reported most commonly in patients with hematologic malignancy, neutropenia, steroid exposure, and other forms of immunocompromised states [2,3,8].

We present a case of fulminant *Rothia* *mucilaginosa* prosthetic valve endocarditis initially presenting as a TTP/DIC mimic with severe thrombocytopenia, microangiopathic features, and rapidly progressive intracranial hemorrhage resulting in a fatal outcome.

## Case presentation

A 60-year-old man with a history of bicuspid aortic valve with severe aortic stenosis status post bioprosthetic aortic valve replacement, opioid use disorder previously treated with methadone, chronic venous insufficiency, chronic hepatitis C infection, and recent right ankle cellulitis initially presented to the emergency department after being found unresponsive by a friend. On arrival, he was encephalopathic and unable to provide additional history. Initial vital signs were notable for temperature 98°F, heart rate 93, blood pressure 119/90, respiratory rate 19, and oxygen saturation 98% on room air. Laboratory evaluation (summarized in Table 1) was notable for profound thrombocytopenia (platelet count 8 × 10³/µL), anemia (hemoglobin 9.4 g/dL), marked leukocytosis (white blood cell count 33.0 × 10³/µL), with neutrophil predominance on differential, including neutrophils 93.0% with an absolute neutrophil count of 31.02 × 10³/µL, lymphocytes 2.0% with an absolute lymphocyte count of 0.66 × 10³/µL, monocytes 3.0% with an absolute monocyte count of 0.99 × 10³/µL, bands 1.0%, and blasts 1.0%, hyperbilirubinemia (total bilirubin 3.5 mg/dL), elevated lactate dehydrogenase (LDH 440 U/L), hypofibrinogenemia (157 mg/dL), and markedly elevated D-dimer (14.2); such findings were concerning for TMA versus DIC in the setting of severe sepsis. Additional laboratory abnormalities included hyponatremia (128 mmol/L), hypoalbuminemia (2.6 g/dL), and preserved renal function with creatinine 0.68 mg/dL. CT head without contrast demonstrated a trace right frontal subarachnoid hemorrhage. Given the concern for intracranial hemorrhage and the need for subspecialty evaluation, the patient was transferred to a tertiary care center for neurosurgical and hematologic evaluation.

**Table 1:** Initial laboratory evaluation on presentation (hospital day 0) Initial laboratory evaluation on presentation demonstrating severe thrombocytopenia, leukocytosis, anemia, elevated hemolysis markers, and coagulation abnormalities concerning for thrombotic microangiopathy and disseminated intravascular coagulation. LDH, lactate dehydrogenase.

Laboratory Test	Result	Reference Range
White blood cell count (×10³/µL)	33.0	4.6-10.2
Hemoglobin (g/dL)	9.4	13.5-17.5
Platelet count (×10³/µL)	8	142-424
Sodium (mmol/L)	128	135-145
LDH (U/L)	440	140-271
Total bilirubin (mg/dL)	3.5	0.3-1.2
Albumin (g/dL)	2.6	3.5-4.8
D-dimer	14.2	<0.50
Fibrinogen (mg/dL)	157	202-543

Given the combination of severe thrombocytopenia (8 × 10³/µL), encephalopathy, schistocytosis (1+ progressing to 2+), worsening anemia (hemoglobin 9.4 g/dL from a prior baseline near 10-12 g/dL), elevated LDH (440 U/L), and low haptoglobin (<30 mg/dL), hematology was consulted for urgent evaluation of suspected TTP. Peripheral blood smear demonstrated circulating schistocytes with additional red blood cell fragmentation abnormalities, including burr cells, elliptocytes, and teardrop cells. Reticulocyte count was elevated (3.8%), and LDH later increased to 1,353 U/L, further supporting microangiopathic hemolytic anemia. Coagulation studies (summarized in Table 2) demonstrated prolonged prothrombin time (PT; 23.1 seconds), international normalized ratio (INR; 1.9), activated partial thromboplastin time (aPTT; 38.8 seconds), persistent hypofibrinogenemia with a nadir fibrinogen of 121 mg/dL, and markedly elevated D-dimer, concerning for concurrent DIC physiology. Hematology initially considered evolving TTP in the differential diagnosis, although the comparatively modest initial LDH elevation and marked leukocytosis with septic presentation raised concern for infection-associated TMA. Subsequently, ADAMTS13 testing was sent while plasmapheresis and intravenous dexamethasone were initiated for empiric treatment of suspected TTP and possible immune thrombocytopenia, respectively. However, serial laboratory monitoring demonstrated that the LDH trend was not as pronounced as typically observed in fulminant TTP. Eventually, plasmapheresis was discontinued due to lower suspicion for primary TTP based on the comparatively modest LDH elevation and trend.

**Table 2:** Hemolysis and coagulation studies Hemolysis and coagulation studies demonstrating microangiopathic hemolytic anemia and consumptive coagulopathy during hospitalization. LDH, lactate dehydrogenase; PT, prothrombin time; INR, international normalized ratio; aPTT, activated partial thromboplastin time.

Study	Result	Reference Range
Schistocytes	1+ to 2+	None
Haptoglobin (mg/dL)	<30	36-195
Peak LDH (U/L)	1,353	140-271
Reticulocyte count (%)	3.80	0.5-2.3
PT (seconds)	23.1	11.9-14.7
INR	1.9	≤1.1
aPTT (seconds)	38.8	23.3-35.3
Lowest fibrinogen (mg/dL)	121	202-543
D-dimer	14.2	<0.50

**Figure 1:**
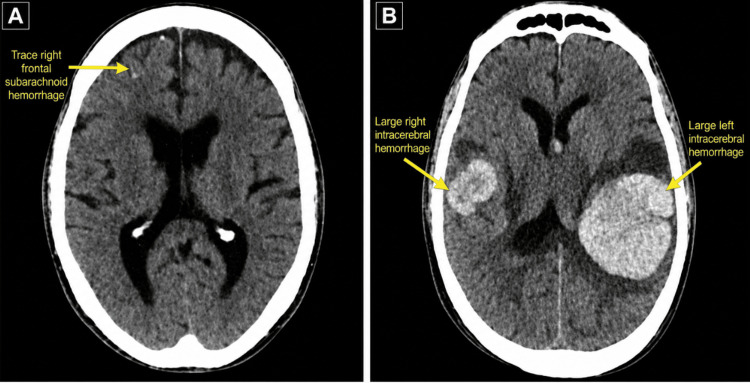
Serial CT head imaging demonstrating progression of intracranial hemorrhage Serial CT head imaging demonstrating progression of intracranial hemorrhage. (A) Trace subarachnoid hemorrhage identified on initial CT imaging on hospital day 0 (yellow arrow). (B) Extensive bilateral intraparenchymal hemorrhages with surrounding edema and mass effect on repeat CT imaging on hospital day 6 (yellow arrows) secondary to hemorrhagic septic emboli from *Rothia mucilaginosa* prosthetic valve endocarditis.

Blood cultures subsequently grew *Rothia mucilaginosa*. Transesophageal echocardiography performed during hospitalization demonstrated bioprosthetic aortic valve stenosis with associated valvular vegetation, supporting prosthetic valve infective endocarditis as the source of bacteremia and septic embolic complications. Infectious disease was consulted, and the patient was treated with intravenous vancomycin and meropenem in addition to ongoing supportive care. Vancomycin was selected for Gram-positive coverage, including *Rothia* species and prosthetic valve endocarditis, while meropenem provided broad empiric coverage in the setting of severe sepsis, clinical deterioration, and pending susceptibility data. Despite treatment, the patient experienced progressive neurologic decline with worsening intracranial hemorrhage, bilateral middle cerebral artery mycotic aneurysms requiring neurointerventional embolization, and multifocal septic embolic complications. Repeat CT neuroimaging later demonstrated extensive bilateral subarachnoid hemorrhage (Figure 1). ADAMTS13 activity later resulted at 7% with negative ADAMTS13 inhibitor testing. Given catastrophic neurologic injury and poor overall prognosis, goals-of-care discussions were held with the family, and the patient was ultimately transitioned to inpatient hospice, where he later passed away.

Overall, the clinical sequence progressed from an initial TTP/DIC mimic with severe thrombocytopenia and intracranial hemorrhage to confirmed *Rothia mucilaginosa* prosthetic valve endocarditis with hemorrhagic septic embolic complications.

## Discussion

This case report highlights a rare and fulminant presentation of *Rothia mucilaginosa* prosthetic valve infective endocarditis complicated by a TMA mimic with features concerning for TTP and DIC. TTP was considered because of severe thrombocytopenia, schistocytosis, hemolysis, neurologic dysfunction, and later severe ADAMTS13 deficiency; DIC was suggested by prolonged coagulation studies, hypofibrinogenemia, and markedly elevated D-dimer; and infection-associated TMA was favored overall given the septic presentation, marked leukocytosis, positive blood cultures, and confirmed prosthetic valve endocarditis. The patient presented with profound thrombocytopenia, microangiopathic hemolytic anemia, altered mental status, and intracranial hemorrhage, ultimately prompting emergent plasmapheresis for presumed TTP prior to recognition of underlying infective endocarditis.

Thrombotic microangiopathies are characterized by endothelial injury resulting in thrombocytopenia, microangiopathic hemolytic anemia, schistocytosis, elevated LDH, decreased haptoglobin, and end-organ dysfunction, particularly involving the neurologic and renal systems [8]. Infection-associated TMAs may closely resemble immune-mediated TTP, creating substantial diagnostic uncertainty during early presentation [1,8]. In a review of systemic infections mimicking TTP, multiple patients initially underwent plasma exchange before infectious etiologies were identified, with many cases associated with poor outcomes and high mortality [1]. Importantly, severe systemic infection and sepsis may produce overlapping features of TTP and DIC through endothelial injury, inflammatory cytokine release, platelet activation, and consumptive coagulopathy. In this case, the coexistence of schistocytosis, severe thrombocytopenia, elevated hemolysis markers, prolonged coagulation studies, and hypofibrinogenemia suggested a mixed TMA-DIC physiology complicating fulminant infection.

Although *Rothia* species are part of the normal oral and upper respiratory tract flora, they are increasingly recognized as clinically significant opportunistic pathogens [2,3]. *Rothia mucilaginosa *infection has been described most commonly in immunocompromised hosts, particularly patients with hematologic malignancy, neutropenia, corticosteroid exposure, indwelling catheters, and prosthetic material [2,3,9]. Of note, the organism possesses biofilm-forming capabilities that may contribute to persistent infection and increased affinity for prosthetic surfaces, including prosthetic heart valves [3,10]. While bacteremia is increasingly recognized, infective endocarditis remains relatively uncommon but is associated with substantial morbidity and mortality [5].

Neurologic complications appear disproportionately frequent in *Rothia* endocarditis. A systematic review of 51 published cases demonstrated neurologic complications in approximately one-third of patients, with embolic phenomena, hemorrhage, and mycotic aneurysm formation contributing significantly to morbidity [5]. Prosthetic valve involvement was identified in 16% of reported cases [5]. Prior reports have described *Rothia mucilaginosa *prosthetic valve endocarditis complicated by cerebral hemorrhage; however, hematologic presentations mimicking TTP or other thrombotic microangiopathies remain poorly characterized [4]. Our case,, therefore, expands the recognized clinical spectrum of Rothia endocarditis by demonstrating severe infection-associated TMA physiology preceding definitive microbiologic diagnosis.

This case also underscores the diagnostic challenge of differentiating primary TTP from secondary infection-associated TMA. Classic TTP is associated with severe ADAMTS13 deficiency and often marked LDH elevation reflecting extensive tissue ischemia and hemolysis [8], whereas secondary TMAs related to infection or sepsis are driven predominantly by systemic endothelial injury, inflammatory activation, and consumptive coagulopathy [1]. In the present case, the comparatively modest initial LDH elevation relative to the severity of thrombocytopenia and neurologic dysfunction introduced diagnostic uncertainty regarding true TTP, although evolving early TTP remained a significant concern. Because untreated TTP carries extremely high mortality, emergent plasma exchange was appropriately initiated while additional diagnostic evaluation was ongoing [8]. Subsequent serial laboratory monitoring demonstrated that the LDH trajectory did not progress as typically expected in fulminant TTP, supporting an alternative diagnosis of infection-associated TMA complicating severe infective endocarditis. Although ADAMTS13 activity later returned severely reduced at 7% with negative inhibitor testing, the overall clinical context, including marked leukocytosis, positive blood cultures, DIC-pattern coagulation abnormalities, and confirmed prosthetic valve endocarditis, favored infection-associated TMA/DIC physiology rather than isolated immune-mediated TTP. Clinicians should therefore maintain a broad differential diagnosis when profound thrombocytopenia and schistocytosis occur in the setting of marked leukocytosis, sepsis physiology, prosthetic cardiac valves, or positive blood cultures. In such scenarios, prompt investigation for underlying infection, including infective endocarditis, remains critical.

Ultimately, our patient experienced catastrophic neurologic deterioration with progression from a trace subarachnoid hemorrhage on presentation to extensive bilateral intracranial hemorrhage despite aggressive multidisciplinary management. This case highlights the aggressive nature of* Rothia mucilaginosa* prosthetic valve endocarditis and emphasizes that infection-associated TMA may closely mimic primary hematologic emergencies such as TTP. Early recognition of underlying infection is essential, particularly in patients with prosthetic valves and evidence of systemic inflammatory response, as delays in diagnosis may contribute to devastating neurologic complications and death.

## Conclusions

*Rothia mucilaginosa* endocarditis is an uncommon but potentially devastating infection that may present with severe hematologic abnormalities mimicking TTP and DIC. This case highlights the diagnostic challenge of distinguishing primary TMA from infection-associated endothelial injury and consumptive coagulopathy, particularly in patients presenting with profound thrombocytopenia, schistocytosis, and neurologic dysfunction. Clinicians should maintain a high index of suspicion for underlying infective endocarditis in patients with sepsis physiology, prosthetic cardiac valves, or positive blood cultures despite an apparent hematologic presentation. Early recognition of the infectious etiology is critical, as delayed diagnosis may contribute to catastrophic neurologic complications and poor clinical outcomes. As this report describes a single case, these findings should be interpreted cautiously, and additional cases are needed to better characterize the relationship between *Rothia mucilaginosa *endocarditis and TMA/DIC-like presentations.
